# Using Serum Amino Acids to Predict Traumatic Brain Injury: A Systematic Approach to Utilize Multiple Biomarkers

**DOI:** 10.3390/ijms21051786

**Published:** 2020-03-05

**Authors:** Marzieh Hajiaghamemar, Todd Kilbaugh, Kristy B. Arbogast, Christina L. Master, Susan S. Margulies

**Affiliations:** 1 Wallace H. Coulter Department of Biomedical Engineering, Georgia Institute of Technology and Emory University, Atlanta, GA 30322, USA; susan.margulies@gatech.edu; 2 Perelman School of Medicine, University of Pennsylvania, Philadelphia, PA 19104, USA; kilbaugh@email.chop.edu (T.K.); arbogast@email.chop.edu (K.B.A.); masterc@email.chop.edu (C.L.M.); 3 Center for Injury Research and Prevention, Children’s Hospital of Philadelphia, Philadelphia, PA 19104, USA; 4 Sports Medicine and Performance Center, Children’s Hospital of Philadelphia, Philadelphia, PA 19104, USA

**Keywords:** head injury, diagnostics, trauma, data science, biomarker, multivariate metrics

## Abstract

Traumatic brain injury (TBI) can cause biochemical and metabolomic alterations in the brain tissue and serum. These alterations can be used for diagnosis and prognosis of TBI. Here, the serum concentrations of seventeen amino acids (AA) were studied for their potential utility as biomarkers of TBI. Twenty-five female, 4-week-old piglets received diffuse (*n* = 13) or focal (*n* = 12) TBI. Blood samples were obtained both pre-injury and at either 24-h or 4-days post-TBI. To find a robust panel of biomarkers, the results of focal and diffuse TBIs were combined and multivariate logistic regression analysis, coupled with the best subset selection technique and repeated k-fold cross-validation method, was used to perform a thorough search of all possible subsets of AAs. The combination of serum glycine, taurine, and ornithine was optimal for TBI diagnosis, with 80% sensitivity and 86% overall prediction rate, and showed excellent TBI diagnostic performance, with 100% sensitivity and 78% overall prediction rate, on a separate validation dataset including four uninjured and five injured animals. We found that combinations of biomarkers outperformed any single biomarker. We propose this 3-AA serum biomarker panel to diagnose mild-to-moderate focal/diffuse TBI. The systematic approaches implemented herein can be used for combining parameters from various TBI assessments to develop/evaluate optimal multi-factorial diagnostic/prognostic TBI metrics.

## 1. Introduction

Traumatic brain injury (TBI) is a leading cause of cognitive and behavioral deficits, disability and death worldwide. TBI results from mechanical forces and rapid motion applied to the brain during sports-related or fall-related head impact events or motor vehicle crashes. These brain biomechanical loads initiate a cascade of short- and long-term cellular and sub-cellular biochemical and metabolomic alterations in the brain which cause primary injury and secondary injury that evolves over time [1] and can lead to long-term neurodegeneration [2]. These biochemical and metabolomic alterations first appear in the brain tissue and then, by crossing a number of barriers, manifest in biofluids such as cerebrospinal fluid (CSF), blood, saliva and urine [1,2,3,4,5,6,7,8,9,10,11,12]. Therefore, biofluids contain valuable information about the occurrence and progression of TBI and thus recently have been explored as a source for potential biomarkers to diagnose TBI, as well as to assess its severity, monitor its progression, predict patient outcomes, and determine the effectiveness of therapeutic interventions [1,4,5,9,10,11,13,14,15,16,17,18,19]. The use of serum biomarkers has greatly contributed to improved diagnostic and therapeutic methods in fields such as hematology, cardiology, oncology, and infectious disease [10]. However, the utility of biofluid biomarkers in the TBI field is in an early stage and requires more study, mainly due to the complexity of the neuropathological process and the heterogeneity of TBI in humans.

A validated and reliable panel of diagnostic biofluid TBI biomarkers can reduce the possible harm of radiation from CT imaging, which is the accepted assessment for diagnosis of moderate to severe TBI in clinical practice today. Further, for milder TBI such as a concussion, where imaging is not often utilized, biofluid biomarkers can serve a critical role in diagnosis and monitoring of recovery. In addition, biofluid biomarkers are effort-independent measures that can avoid the challenges that exist with many clinical semi-quantifiable outcome measures — such as the vestibular/ocular-motor screening (VOMS) [20] and sport concussion assessment tool 5 (SCAT5) [21]— which are commonly used in TBI. In addition, biofluid biomarkers can be explored in both animals and humans in a preclinical-clinical translational manner to advance the diagnosis, prognosis and treatment of TBI. The translatable aspect of biofluid biomarkers highlights the role of preclinical studies in reducing the heterogeneity seen in human TBI.

Over the last few years, several biofluid biomarkers such as ubiquitin carboxy-terminal hydrolase L1 (UCH-L1), neuron-specific enolase (NSE), S100β, glial fibrillary acidic protein (GFAP), neurofilament light chain (e.g., NF-L) and tau have been studied as diagnostic and prognostic markers of TBI [1,13,15,22,23]. In addition to these biomarkers that are directly associated with primary neuronal, glial, or axonal damage due to TBI, the concentration levels of amino acids and other metabolite markers in CSF, serum, and even urine, have been shown to be significantly different in patients with TBI in comparison to non-TBI subjects in clinical studies [5,8,12,16,19] and are altered following TBI in comparison to pre-injury conditions in pre-clinical studies [3,6,7,8]. The metabolite biomarkers have been shown to be related to secondary injury and associated with acute to chronic neuropathological sequelae, neurodegeneration, cognitive impairments, as well as energy deficits and damages in the brain tissue following TBI [8,19]. Studies suggested that metabolite changes have great potential to be used as diagnostic and prognostic biomarkers for TBI and TBI-induced neurodegeneration [1,2,3,8,16,17,19,24,25].

Among metabolite biomarkers, amino acids are particularly important as they play a key role in neuronal circuitry development and maintenance and neuronal growth and survival in the brain [26]. Most of the studies that have explored amino acids as potential biomarkers of TBI used CSF as the biofluid source and focused on severe TBI. For example, the CSF concentrations of the excitatory amino acids, glutamate and aspartate, within 7 days post-TBI were found to be higher in patients with severe TBI than healthy controls and higher in TBI patients with worse outcomes [17]. In another study, the CSF levels of glutamine, glycine, serine, and histidine were found to be significantly higher in patients with subarachnoid hemorrhage (SAH) compared to control patients [9]. In another study, the CSF levels of glutamate and lactate within 2–4 h post-severe TBI were found to be significantly higher in non-survival than survival patients [16]. While these studies provide useful insight into the metabolic disruption caused by TBI and the utility of metabolites as biomarkers of TBI, sampling CSF is not a clinically feasible practice in patients with mild TBI. In mild TBI, serum biofluid biomarkers are more acceptable and less invasive than CSF and need to cross fewer barriers compared to saliva or urine biofluids. Thus, much of the research today focuses on serum biomarkers for TBI.

Only a few studies, however, have investigated the alterations of serum metabolite levels following TBI and examined them as potential diagnostic and monitoring markers of mild TBI. For example, a study examined the serum levels of branched chain amino acids (BCAA), leucine, isoleucine and valine, and found them to be lower in patients with mild TBI compared to non-TBI controls, with lower values indicating more severe injury [24]. In that study, the serum level of BCAAs was found to be correlated with the magnitude of increased intracranial pressure (ICP) as well [24]. Another study used a panel of nine serine-related metabolites collected from the serum of patients within three days post-TBI to discriminate between healthy subjects and TBI patients with and without cognitive impairment [19].

Given the variety of metabolomic biomarkers and their role in the short- and long-term neuropathological outcomes/deficits post-TBI, more studies are needed to evaluate serum metabolites as potential biomarkers of TBI. The small number of studies that previously examined serum metabolites as TBI biomarkers in the literature may be due to several factors. First, there are different mechanisms for transporting amino acids into the brain crossing the blood brain barrier (BBB) [27]. Some of these mechanisms may become damaged in TBI and thus some biomarkers may not be measurable in serum [10]. Additionally, metabolite concentrations in the blood are lower than they are in the brain and/or CSF and require more sensitive assays to accurately measure their concentrations. The low concentrations in blood can also increase the variability in the measurements. With the recent advancement in more precise metabolomic assays [28], more studies are expected to be conducted to explore serum metabolite biomarkers of TBI in the near future.

In this study, we used the serum concentration of amino acids (AA) from pre-clinical focal and diffuse juvenile pig models of TBI to determine which combination of amino acids could serve as an acute diagnostic biomarker for mild-to-moderate TBI using a systematic multivariate approach. We also investigated the temporal profile of each individual AA in serum over the first 4 days post-TBI. The systematic approach used in this study to develop and evaluate an optimal multivariate AA panel could also be used for combining parameters from various TBI assessment techniques such as imaging, electroencephalography (EEG), clinical measures including objective balance and gait tests, and quantitative oculomotor and vestibular tests to develop and evaluate optimal multi-factorial diagnostic/prognostic TBI metrics..

## 2. Results

**Figure 1:**
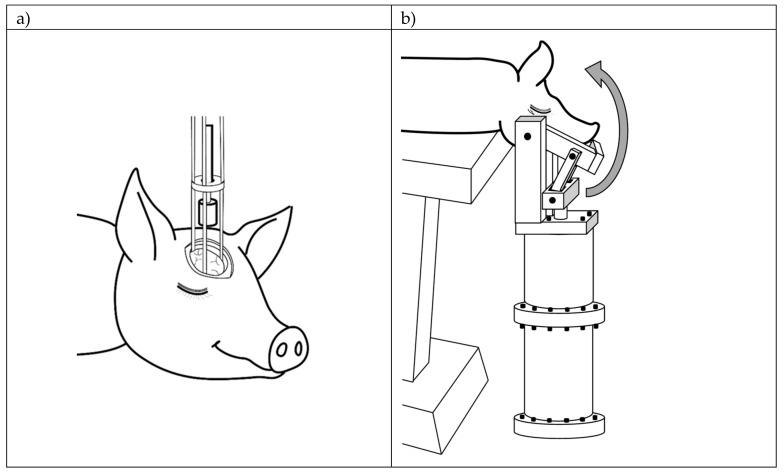
Schematic representation of (**a**) controlled cortical impact (CCI) device and (**b**) rapid non-impact head rotation (RNR) pneumatic device.

**Figure 2:**
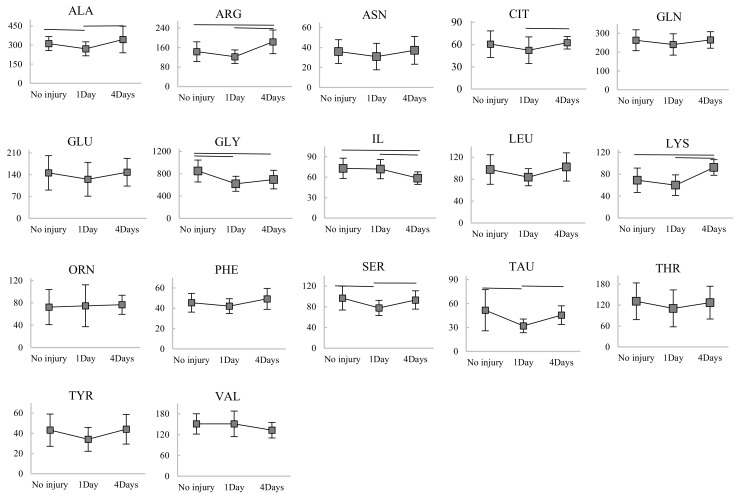
Alteration of serum concentrations (in nm/mL) of amino acids over time after TBI (in the Development dataset). Statistically significant differences in the concentrations of amino acids between time points are noted with horizontal lines.

In this study, two standardized and validated models of TBI in piglets, rapid non-impact head rotation (RNR) and controlled cortical impact (CCI) (shown in Figure 1) [19] which represent diffuse and focal TBI, respectively, were used. Twenty-five animals received a diffuse TBI (RNR, *n* = 13) or a focal TBI (CCI, *n* = 12) and blood samples were obtained before and either 24 h after (CCI, *n* = 8 and RNR, *n* = 8) or 4 days after (*n* = 4 CCI and *n* = 5 RNR) injury, resulting in 50 blood samples hereafter referred to as the **Development Dataset** (Table 1). Blood samples obtained before injury were used as uninjured controls. The concentrations of seventeen out of twenty AAs including alanine (ALA), arginine (ARG), asparagine (ASN), citrulline (CIT), glutamine (GLN), glutamate (GLU), glycine (GLY), isoleucine (IL), leucine (LEU), lysine (LYS), ornithine (ORN), phenylalanine (PHE), serine (SER), taurine (TAU), threonine (THR), tyrosine (TYR), and valine (VAL) were measured in the serum samples. The concentrations of the other three AAs, including methionine, tryptophan and aspartate, could not be measured in all the samples, and thus, these three AAs were excluded from further analysis. The results of serum samples from both RNR and CCI injuries were grouped together to develop a diagnostic biomarker for early (≤4 days) heterogeneous TBI, inclusive of focal and diffuse types. First, the changes in serum concentration biomarkers for all AA over time (from pre-injury to 24 h and 4 days following TBI) were evaluated in the development dataset to determine whether significant serum AA alterations developed over time post-TBI (Figure 2). Only glycine, serine, taurine and alanine concentrations were significantly decreased at 24-h post-injury compared with pre-injury (Mann–Whitney test, *p* < 0.05). Glycine levels continued to remain depressed significantly at 4-days post-TBI while serine, taurine and alanine returned to pre-injury levels at 4-days. In addition, significant increases in arginine and lysine levels and a significant decrease in isoleucine at 4-days post-TBI compared with pre-injury were observed. 

**Figure 3:**
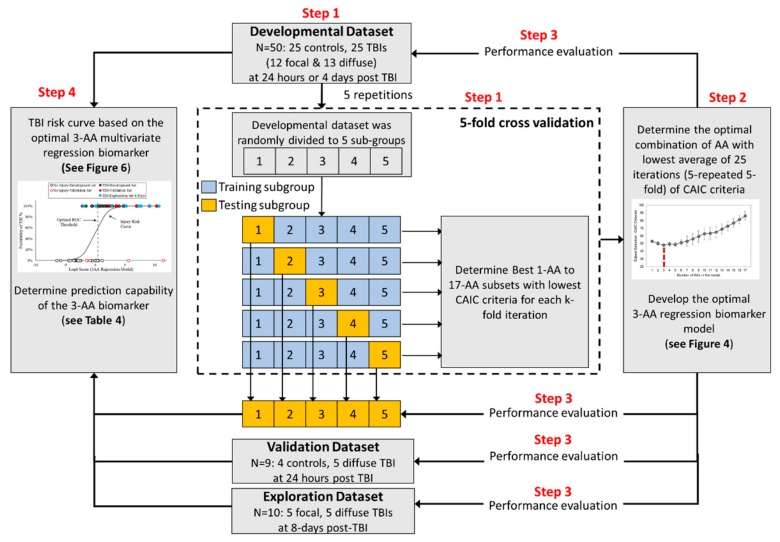
A schematic summarizing the workflow of the statistical process used in this study for development and evaluation of the optimal multivariate amino acid biomarker.

**Figure 4:**
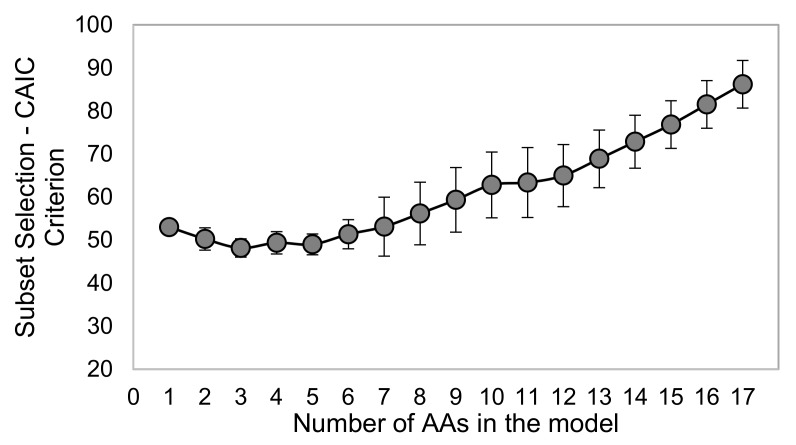
The results of best variable selection procedure. The average and standard deviation of CAIC criterion of the 1- to 17-AA best subset models over all 25 iterations of five repeated five-fold CV are shown.

The development dataset was next used to develop a robust multivariate biomarker metric with high sensitivity and specificity for diagnosis/prediction of TBI by finding an optimal combination of these amino acids, taking into consideration the interactions between various AA levels following TBI. A rigorous process for selecting the optimal variates for such a metric was performed as described in detail later in the Methods and Materials section. A schematic summarizing the workflow of the statistical process used in this study for development and evaluation of the optimal multivariate biomarker is shown in Figure 3. Using the repeated 5-fold cross validation (CV) procedure, a thorough search of all possible subsets of AAs was performed to determine the best subset of AAs with smallest values of Consistent Akaike’s information criterion (CAIC) [29] for groups of 1- to 17-AA at each iteration. The average and standard deviation of CAIC of the 1- to 17-AA best subset models over all 25 iterations are shown in Figure 4. The results of this process suggested that the 3-AA subset was the best model with the lowest average value of CAIC criterion in the repeated k-fold CV. In 24 out of 25 iterations of the repeated 5-fold CV procedure, glycine, taurine, and ornithine were selected as the best 3-AA subset.

**Figure 5:**
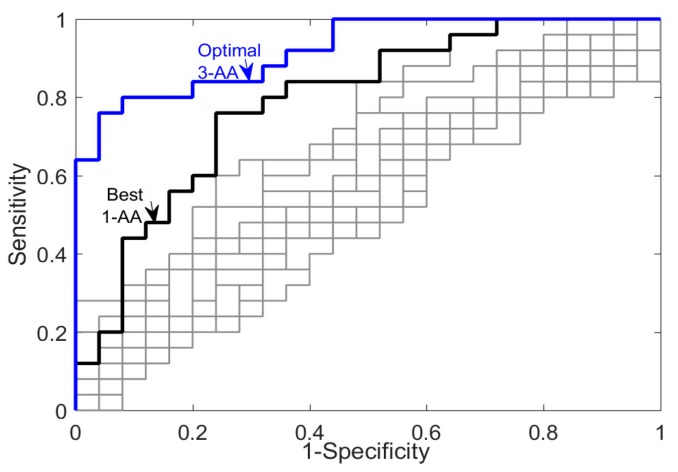
The ROC curves for the optimal multivariate amino acid regression biomarker and all seventeen amino acids as univariate biomarkers.

Logistic regression model (Logit) was developed using the 3-AA subsets from the development dataset. The details of this logit regression model are given in Table 2. Each of these 3 AAs (glycine, taurine, ornithine) significantly contributed to the regression model in discriminating between injured and uninjured samples (*p* < 0.05). The discriminatory power, evaluated by area under the ROC curve (AUC), was much higher for the 3-AA biomarker regression model (0.9216) than for any of the 17 AAs as a univariate AA biomarker (AUC = 0.5216-0.7204). The univariate AA biomarkers with highest AUC were glycine (AUC = 0.7902), serine (AUC = 0.6794), and taurine (AUC = 0.6544). Furthermore, it should be noted that in all 25 iterations, glycine was determined as the best 1-AA subset. The ROC curves for the optimal 3-AA multivariate biomarker and all the AAs as univariate biomarkers are shown in Figure 5. The AUC and optimal ROC threshold value of the optimal 3-AA model as well as each of univariate AA biomarker are given in Table 3.

**Figure 6:**
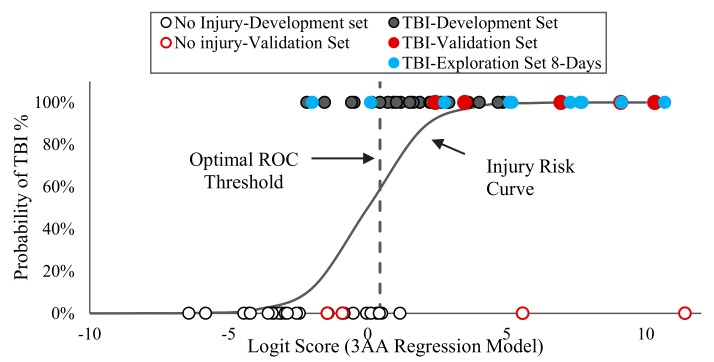
The TBI risk curve based on the optimal multivariate regression biomarker using combination of glycine, taurine, and ornithine serum concentrations and the optimal ROC threshold value (dashed line) of the optimal 3-AA model are shown. The optimal 3-AA regression model was applied and the logit scores for the Development Dataset, Validation Dataset, and Exploration Dataset were calculated.

A probability risk curve, P as described in the Methods and Materials section, was also developed based on this optimal 3-AA regression biomarker as shown in Figure 6. This optimal 3-AA regression model was applied to the development dataset as well as to a separate dataset of additional animals, the Validation Dataset, and the corresponding logit values/scores were calculated and shown in Figure 6. The **Validation Dataset** (Table 1) consisted of four sham and five piglets which underwent RNR TBI experiments and their blood and brain tissue samples were collected at 24 h post-TBI (RNR) or at 8 days post-sham procedure. This Validation Dataset (*n* = 9) were used to evaluate the prediction capability of the optimal multivariate 3-AA biomarker. A third separate dataset of additional animals was defined as the **Exploration Dataset** (Table 1), composed of ten piglets which received diffuse (RNR, *n* = 5) or focal (CCI, *n* = 5) TBI and blood was obtained at 8 days post-TBI. Serum levels of AAs measured in this dataset were used to evaluate the diagnostic prediction capability of the optimal 3-AA biomarker to diagnose TBI at a later time point post-injury.

The prediction capability measures including sensitivity, specificity and overall accuracy rate were calculated for these three datasets (development, validation, and exploration datasets) and the results are given in Table 4. The prediction capability was also evaluated through repeated 5-fold CV for the development set. This optimal 3-AA model not only showed high prediction capability (S = 80%, SP = 92%, PAR = 86%, repeated 5-fold CV: S = 80%, SP = 92%, PAR = 86%) on the development set but also high sensitivity (S = 100%) in identifying TBI cases from the validation dataset. This model also showed 80% sensitivity in recognizing TBI from the Exploration Dataset comprised of data from a later time point after injury (8 days post-TBI). All these measures confirmed the high prediction capability of the optimal serum GLY-TAU-ORN logit model as TBI diagnostic biomarker.

**Figure 7:**
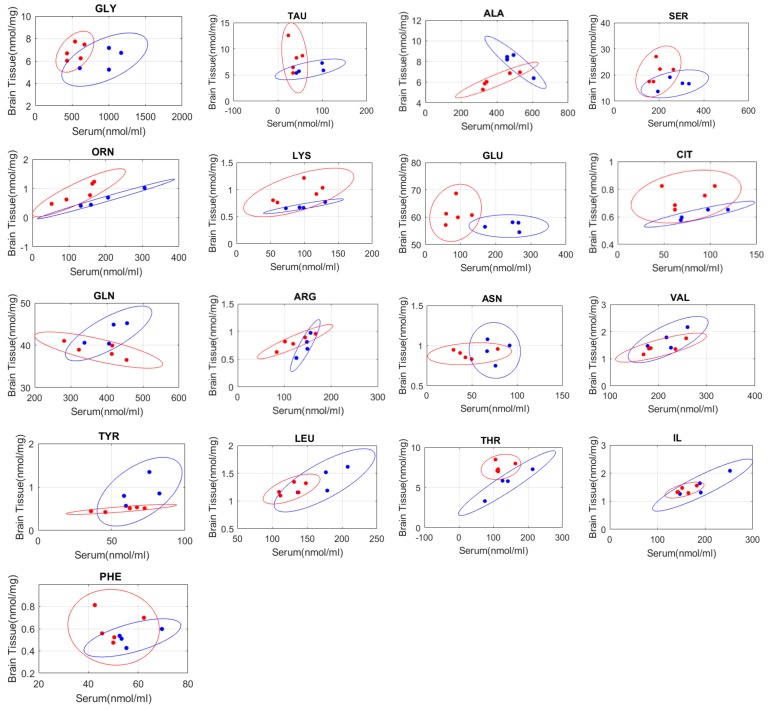
The correlations between serum and brain tissue concentrations of seventeen amino acids and the corresponding 95% confidence ellipses for both healthy/sham (blue markers) and 24 h post-TBI (red markers) groups were shown to explore possible alterations of tissue-serum amino acid concentrations following TBI. These results are based on the Validation Dataset that includes four sham and five TBI samples.

In addition, we harvested cortical tissues from the nine piglets that comprised the validation dataset. The brain tissue was extracted at sacrifice, flash frozen, and AA levels in the homogenate tissue were measured. These measurements were used to investigate possible correlation/interaction between serum and brain tissue amino acid concentrations and to explore possible alterations of these correlations/interactions following TBI. Scatter plots of the relations between each AA concentration in blood and tissue with corresponding 95% confidence ellipse for sham and injury groups from the development dataset are illustrated in Figure 7. Pearson correlation analysis was performed for these data and compared between sham and injury groups for each AA. Correlation coefficients (R^2^) are given in Table 5. These plots illustrate how relationships between amino acids in the brain tissue and blood might be affected due to TBI. For instance, glutamine tissue-sham correlation coefficient (R^2^) is −0.76 for sham and 0.78 for injured group which indicates good correlation between tissue and serum level of glutamine in both injury and sham groups. However, these correlations are in the opposite direction, indicating that glutamine may be increased in brain tissue while decreased in blood as the result of TBI. Reduction of glutamine concentration in serum at 24 h post-TBI was also observed in our development dataset when compared to pre- and 24 h post-TBI results as shown in Figure 2. This opposite alteration might be a reflection of an exchange of glutamine between brain tissue and blood, which might be attributed to a high demand for glutamine in the brain tissue following TBI which caused a reduction of glutamine in serum.

## 3. Discussion

### 3.1. Systematic Approach to Develop and Validate a Multivariate Biomarker of TBI

In this study, a systematic approach was used to find the simplest and most robust combination of amino acids with the highest prediction capability as a panel of biomarkers for the acute diagnosis of TBI. A logistic regression model was developed using the optimal combination of amino acids identified in the first step and prediction capability of this model was evaluated rigorously in several ways. Below, the advantages of the systematic approach used in this study for variable selection and biomarker model development and evaluation are discussed.

Serum biomarkers can be used either individually, as a univariate marker, or combined, as a multivariate panel of markers, to predict the likelihood of the presence and severity of TBI. Due to the heterogenous and multi-faceted nature of TBI, multi-variate biomarkers have advantages over univariate biomarkers for the prediction of TBI and its outcome. Therefore, recently, more studies are focusing on combining multiple biomarkers to capture different metabolic processes, neuropathological sequelae, molecular interactions, and deficits of TBI [3,13,19,23,30,31].

An important consideration in either uni- or multi-variate biomarker discovery is the reliability of the process used to develop the biomarker model and evaluate its prediction performance accuracy in facing unseen data from new subjects. Most TBI studies in the literature have examined one or a number of biomarkers individually as univariate TBI biomarker on datasets including TBI and non-TBI/healthy control groups, and the prediction performance of each biomarker was assessed individually using ROC curve analysis on the same datasets without any independent and/or cross-validation of the prediction performance on separate evaluation/testing dataset(s) [15,16]. This approach, in which all data are used for both development and evaluation of the biomarker model, is called the “all in” approach. While the univariate biomarker studies using the “all in” approach have led to many findings in the biomarker TBI field and have laid a good foundation to demonstrate that the serum biomarkers can be used as diagnostic and prognostic tools for TBI, multivariate data analysis, advanced supervised/unsupervised classification approaches, and cross validation techniques can enhance the predictability, performance, and reliability of TBI biomarkers, as well as the processes used to identify them.

One important factor for consideration in biomarker data analysis are the challenges posed by the “all in” approach. This approach may cause overfitting, dependency of the model to the development dataset, and biased assessment of the prediction performance, especially in multivariate analysis with small size datasets. Repeated random training–testing splitting and repeated k-fold cross-validation techniques, in which data are repeatedly split into independent and non-overlapping development and validation datasets, can be used instead of “all in” approach to prevent overfitting and provide more realistic assessment of the accuracy performance of the biomarker models in diagnosing and predicting the outcomes of new patient(s)/subject(s) [32]. Repeated k-fold cross-validation has superiority over repeated random training–testing splitting because the number of times that each data point is used in the training and testing datasets is the same for all data points. These methods are common in data science but have not been used widely in TBI biomarker studies. In this study, we used repeated k-fold cross-validation technique to prevent overfitting and develop a robust multivariate biomarker.

Another important process in biomarker data analysis is choosing the appropriate supervised/unsupervised classifier algorithm. Studies show that supervised classifier algorithms perform better than unsupervised methods when development of predictive models of injury and future outcome is the goal rather than identification of hidden patterns within the dataset [33]. Some supervised algorithms that have been used previously to construct classification models between different injury and non-injury classes in biomarker studies include linear discriminant analysis (LDA) [14], logistic regression analysis (Logit) [22], and k-nearest-neighbors (KNN) [34]. LDA and logit algorithms are more common and advantageous than KNN algorithms because they are easier to be implemented and interpreted. In addition, there is a high chance of overfitting and dependency to the training dataset with a KNN algorithm because the importance of variables is not systematically weighted in this approach which may negatively affect the classifier reliability, especially when facing new data in high dimensional cases with dispersed variables [35]. Logit, described in details in the Methods and Materials section, has superiority over LDA because as opposed to LDA, logit does not require normal Gaussian distribution of variables and provides relatively robust results regardless of variable distributions [36]. Moreover, the LDA algorithm has some other drawbacks that logit does not have: LDA is sensitive to outliers and cannot accommodate non-linearly separable classes [37]. In this study, we used logit algorithm for multivariate biomarker development because some of the amino acid variates did not have normal Gaussian distribution.

In the multivariate biomarker development process, selecting proper dimensional reduction or variable selection technique is also an important factor for consideration to select the most informative and parsimonious list of variables, thus increasing the stability/reliability while avoiding overfitting. Several variable selection methods, common in the data science field, are backward stepwise elimination, forward stepwise selection, and best subset selection [38,39,40]. Backward stepwise elimination starts with the full model with all variables included and sequentially deletes the predictor that has the least impact on the fit starting with variables with the largest p-value. Forward stepwise selection starts with one variable with the smallest p-value and adds one variable at each iteration until no significant discriminatory power/predictive capability improvement is obtained by adding an additional variable. Best subset selection, as described in detail in the Methods and Materials section, is performed by exhaustively searching all combinations of available variables and different selection criteria are used to pick the optimal subset resulting in highest discriminatory power and predictive accuracy performance. Although best subset selection requires high computational cost, it is preferred over the stepwise selection approaches to prevent possible bias since stepwise approaches select variables sequentially and only explore a small fraction of the possible number of subsets available and can be biased by the starting set and order of parameter addition or subtraction [38,39,40]. In this study, we used the best subset selection approach to determine an optimal, robust, and reliable subset of variables in the multivariate biomarker and prevent possible bias.

In this study, with the goal of developing a reliable and robust multivariate panel of biomarkers, multivariate logistic regression analysis, coupled with repeated k-fold CV and the best-subset selection technique, was used and the combination of serum concentrations of glycine, taurine, and ornithine was identified consistently (24 out of 25 iterations) as the optimal subset of amino acids for prediction of TBI. The logistic regression model developed using these three serum AA levels showed an excellent TBI prediction capability, with 80% sensitivity, 92% specificity, and 86% overall accurate prediction rate for the development cohort and 100% sensitivity and 78% overall accuracy rate for a separate validation cohort. This 3-AA model also discriminated TBI at longer time periods post-TBI (8 days post-TBI) with 80% sensitivity. The high TBI prediction capability of the model on Development Dataset, Validation Dataset, and Exploration Dataset (shown in Table 4) demonstrates that this GLY-TAU-ORN regression biomarker model is a robust, parsimonious, and optimal TBI AA biomarker model for both focal and diffuse TBI. The AUC for glycine, taurine, ornithine as univariate AA biomarkers ranged from 0.5216–0.7204, while AUC for the panel of GLY-TAU-ORN is 0.9216. This result shows the advantage of multivariate analysis over univariate relationships in biomarker discovery. Among the optimal 3 AAs selected, only glycine and taurine were significantly changed following TBI and had the first and third highest discriminatory power compared to the other AAs. Interestingly, ornithine did not significantly change post-TBI and had lowest discriminatory power, however, when combined into a multivariate model, it was selected as one of the best three AA biomarkers for diagnosis of TBI.

Overall, the results suggested that alteration in multiple amino acids can be used to discriminate uninjured from injured conditions and suggests that this multivariate panel of amino acids can be used as an acute diagnostic tool for TBI. In addition, the systematic approach that was used in this study to find the optimal combination of biomarkers and to rigorously evaluate the prediction capability of the model can be implemented in other multi-modal biomarker studies. Although the 3-AA biomarker logit model developed in this study may not be directly translatable to the clinical setting due to possible AA serum concentrations differences between humans and piglets, the combination of these 3 AAs determined herein could also be explored in humans.

### 3.2. Alteration of Amino Acids Concentration in Blood and Tissue Following TBI

Among the 17 serum AAs examined in this study, only glycine was significantly decreased post injury at all time points (from 850 ± 200 nmol/mL for pre-injury/healthy to 620 ± 138 nmol/mL at 24 h post-TBI, 696 ± 167 nmol/mL at 4 days post TBI and 627 ± 224 nmol/mL 8 days post TBI). Depression of serum glycine level following mild and severe TBI was also found in humans [41]. Glycine is present in high concentrations within the central nervous system and serves as both an inhibitory and excitatory neurotransmitter [42]. In this study, it showed higher concentration in serum compared to the other AAs examined (Figure 2). Glycine was the best 1-AA subset in all iterations of repeated k-fold CV variable selection process, with the highest TBI prediction capability and discriminatory power when AAs were examined as univariate biomarkers (Figure 5). The long-time TBI-induced alteration behavior, higher TBI discriminatory power and higher concentrations of glycine in blood compared to the other AAs make glycine a promising biomarker candidate for acute diagnosis and maybe even monitoring of TBI. Interestingly, as opposed to the continuous depression of glycine serum concentration that was observed following TBI, the glycine tissue concentrations, in the small sample size tested, were higher in the TBI group than the healthy group. The increased levels of glycine in brain tissue following TBI has also been observed in previous studies [5,18,43,44]. Since glycine is among the small neutral amino acids that can easily cross the blood-brain barrier, the decreased serum glycine and increased brain tissue glycine following TBI might reflect the TBI-induced oxidative stress pathology in the brain tissue and the role of glycine in mitigating such a pathology. Oxidative stress is known as one of the key pathologic mechanisms of secondary injury following TBI [45]. It represents a mismatch between oxidant and antioxidant agents caused by the excessive glutamate and activated glutamate receptors following TBI and can result in neuronal cell death and dysfunction [46]. As an antioxidant agent, glycine along with cytosine, is the precursor for the synthesis of glutathione, which is the major antioxidant molecule in the body. Therefore, glycine can contribute to reducing oxidative stress following TBI, and is believed to have neuroprotective capability, with the capability of reducing neuronal death and attenuating brain injury in intracerebral hemorrhage [47].

Serine, taurine and alanine concentrations were also significantly decreased at 24-h post TBI but return to normal/pre-injury values at 4 days post-TBI. Glycine, serine, and alanine are the three critical amino acids, involved in glucagon production, involved in glycogen metabolism which is triggered by TBI [48]. Depression of the plasma level of serine, alanine, glycine and taurine at 4hrs and 24hrs post-TBI was also reported by another study conducted on mice using weight drop impact (WDI) injury model [3]. WDI model can induce focal cerebral contusion and diffuse axonal injury. In our study, these 4 AAs (glycine, taurine, alanine, and serine) showed higher concentration levels in brain tissue and a larger ratio of tissue to serum concentration levels for TBI group compared to sham/healthy in the small sample size examined (Figure 7). Similar changes in the tissue-serum AA ratios/correlations were observed for ornithine, glutamate, lysine, and citrulline, illustrating alterations in opposite directions of these amino acid concentration levels in serum and brain tissue following TBI (Figure 7). The elevated concentrations of glycine, taurine, glutamate, alanine, serine, and/or citrulline in brain tissue following diffuse TBI [43] and focal TBI [44,49] have also been reported by other pre-clinical studies. In clinical studies, the CSF concentrations of glutamate, glycine, and serine have been reported to be elevated following severe TBI and the increased CSF glutamate and glycine have been shown to be associated with poor outcomes [5,18]. The opposite direction of the alterations of these AAs in serum and brain or CSF following TBI, observed in our study and previous studies in the literature [5,18,43,44,49], raise the question of how the alteration of AA levels in brain tissue following TBI can affect the AA levels in serum as a reflection of TBI. Although the sample size of the Validation Dataset used herein for exploring AA levels in tissue was too small for a general conclusion, one explanation for the opposite direction of alteration of AAs in serum and brain or CSF could be the high demand for these AAs in the brain tissue after injury and thus the exchange of these AAs between blood, CSF and brain. In addition to the antioxidant role of glycine, which might explain the increased level of glycine in the brain tissue and CSF and decreased level of it in serum following TBI, glycine and serine interact with glutamate receptors. The increased tissue concentration of glycine and serine in the brain tissue might occur to mediate the glutamate-excitotoxicity, mitochondrial dysfunction, and neuronal damage caused by the elevated glutamate and over-activation of glutamate receptors following TBI [43,50,51]. The higher levels of taurine in brain tissue and decreased levels in serum might also occur to counteract the TBI-induced glutamate-mediated excitotoxic cell death, because taurine regulates the ability of mitochondria to reduce, guard or buffer the intracellular calcium during glutamate excitotoxicity [52].

### 3.3. Limitations and Future Directions

Several limitations with the study should be acknowledged. In this study, serum samples were collected at limited time points, either at 24 h or 4 days post-TBI. Future studies should focus on more frequent and serial sampling as well as sampling at later timepoints following TBI. In addition, as mentioned previously, the number of data used for exploring AA levels in the brain tissue was too small. Future studies should examine larger dataset to better elucidate alterations of correlations/interactions between serum and brain tissue amino acid concentrations following TBI and determine possible underlying explanations for that. Furthermore, in this study, preclinical piglet models of TBI were used and while there are advantages of preclinical studies for 1) reducing the heterogeneity seen in human TBI; 2) focusing on one TBI mechanism, type and severity at a time; 3) exploring the underlying reasons for specific pathological, functional, or behavioral deficits; 4) accelerating development of diagnostic and prognostic tools as well as neuroprotective and therapeutic interventions for various TBI types and severities, there are also limitations and challenges with translation of the outcomes to clinical application given possible AA serum concentrations differences between humans and piglets. Future studies could use our methods to analyze serum concentrations of AAs in humans with TBI.

## 4. Materials and Methods

Two animal models of TBI, RNR and CCI, were used. The study was limited to female, 4-week-old piglets (8.2 ± 1.2 kg) to eliminate potential variance due to gender and age that may influence brain injury outcomes and serum biomarkers [20]. The details of the animal preparation, monitoring, and procedures of these two TBI models are described in the next sub-sections and more details can be found in previous publications from our group [6,7,53]. The protocols for the animal models used in this study were approved annually by the Institutional Animal Care and Use Committee of the University of Pennsylvania (protocol numbers 803401 and 801888), where the experiments were performed, and all procedures were carried out in accordance with the guidelines and regulations for the Care and Use of Laboratory Animals of the National Institutes of Health. Blood samples were obtained under anesthesia before injury and also after the injury at the time of euthanasia (different time points post injury for different groups of piglets) from peripheral veins using 3cc syringe attached to intravenous (IV) catheter. This study consisted of three datasets: development, validation, and exploration (more details about these datasets can be found in Table 1). The concentrations of seventeen AAs were determined via HPLC with an Agilent 1260 Infinity LC system utilizing pre-column derivatization with o-phthalaldehyde [28]. The details of the protocol used for measurement of AA levels in the serum and tissue are published elsewhere [28]. These measurements were performed in the Children’s Hospital of Philadelphia (CHOP) Metabolomic Core. Brain tissues were collected for either perfusion fixed with formalin for histological analysis or fresh for metabolite biomarker measurements. To harvest the fixed brain tissue, piglets were given an overdose of pentobarbital and were perfused post euthanasia and fixed brain were collected for pathology analysis. To harvest the fresh brain tissue, the piglets were euthanized with an overdose of pentobarbital (150 mg/kg) via IV or intracoronary (IC) after a terminal surgery procedure and fresh and unfixed brains were collected. For collection of fresh brain tissue, craniotomy was performed and left frontal cortex was resected, blood and vasculature were dissected, and brain tissue was homogenized. Then, the brain tissue samples were covered with optimal cutting temperature compound (OCT compound), kept in OCT for 3-5 min, frozen immediately after that, and stored at −80 °C till the AA measurement procedure. The brain tissue from right cortex were used for measurements of AAs. The collection of fresh and unfixed brain and measurements of AA levels in the brain tissue were only performed for the Validation Dataset (*n* = 9 including 5 RNR TBIs and 4 shams). Both of the injury models used in this study have previously showed to cause mitochondrial dysfunction [6,7] and brain damage such as brain contusion, intracranial hemorrhage, and/or diffuse axonal damage as determined by MRI imaging analysis and pathology analysis [32,53,54,55]. However, the animals typically recovered quickly, reached the baseline physiology within 1–5 h post injury. Anxiety and somnolence were observed in these animals over the first two days following TBI but, as opposed to severe TBI condition, the animals did not require additional clinical care after recovering from anesthesia [6,21]. Therefore, the severity of the TBI experiments is classified as mild to moderate based on the clinical severity classifications by comparing the sustained deficits in these piglets to the ones in humans [6,21,24].

### 4.1. Animal Preparation

Piglets were fasted starting 12-h prior to the anesthesia procedure (either for injury, sham, or euthanasia procedure). On the day of experiment, piglets were premedicated with intramuscular injection of ketamine (20 mg/kg IM) and xylazine (2 mg/kg IM) prior to anesthesia. Then, anesthesia was induced with the inhalation of 4% isoflurane via snout mask, until loss of response to reflexive pinch stimuli, and maintained with inhalation of 1% isoflurane. When the desired depth of anesthesia was achieved the animals were intubated and maintained on 100% oxygen while breathing spontaneously. Mechanical ventilation was used when necessary to maintain peripheral saturations greater than 94% and normocarbia as defined as End Tidal Carbon Dioxide (ETCO2) before injury, otherwise piglets maintained spontaneous ventilation. Body temperature was kept constant between 36 and 38 °C using a circulating water heating pad and monitored via a rectal probe. Vital signs, blood pressure, oxygen saturation, heart rate, respiratory rate, and ETCO2 were continuously monitored throughout the experiments (VetCap model 2050081; SDI, Waukesha, WI). Furthermore, Buprenorphine (0.02 mg/kg) was delivered intramuscularly for analgesia prior to injury. It should be noted that except the fasting period prior to the anesthesia procedure (prior to the injury/sham and the euthanasia procedures), piglets had normal feeding and received standard pig food pellets. Marshmallow fluff occasionally might be used as reward. No water fasting was performed at any time during the study and no food reward was used during the fasting period.

### 4.2. Controlled Cortical Impact TBI Model

A well-established CCI TBI piglet model [6,53,56], as shown in Figure 1, was used to produce focal cortical contusion utilizing a validated skull-mounted, spring-loaded blunt indentation device to create a rapid (4 ms) displacement of the cortical surface with no inertial motion of the head. This injury mechanism produces focal contusion, with underlying white matter damage, decreased cerebral blood flow (CBF) and somatosensory dysfunction. As previously described [6,53,56], a craniectomy, 1 cm larger than the indentor tip, was performed on the right coronal suture of the animal under anesthesia and the dura was opened to expose the cortical surface. Then the indentation device was firmly attached to the skull with screw fixation and the spring-loaded indentor tip was ejected over 4 msec to a depth of 0.7 cm of the cortical rostral gyrus and then removed. The cortical surface was irrigated, the dura was reapproximated, and the scalp was sutured closed and sealed with Dermabond. Immediately after the injury procedure, animals were recovered from anesthesia and extubated when animals met the following criteria: return of pinch reflex; spontaneously breathing and able to maintain oxygenation and ventilation; normotensive; stable heart and respiratory rates. After extubation, animals were returned to the animal housing facility when they met the following criteria: vocalization without squealing, steady ambulation, no aggression or avoidance behavior, no piloerection, and proper feeding and drinking behaviors [6,53,56].

### 4.3. Rapid Non-Impact Rotational TBI Model

An RNR TBI model, as shown in Figure 1, utilizing a well-characterized rotational acceleration device [1,7,32,53,55] was used to produce a single rapid sagittal head rotation causing purely inertial diffuse axonal injury. This injury mechanism produces unconsciousness, sustained cognitive dysfunction, decreased CBF, bilateral diffuse axonal and hemorrhagic injury. As described previously [1,7,32,53,55], while the animal is under anesthesia, the head is secured to the inertial injury apparatus by a snout clamp. Immediately prior to injury, anesthesia, supplemental oxygen, and mechanical ventilation were withdrawn, so the piglet could breathe spontaneously on room air. Activation of the inertial loading device rapidly rotates the head through a 60-degree sagittal arc in 10 to 40 milliseconds. Following injury, the piglet was removed from the injury device, supplemental oxygen was resumed as needed, isoflurane was discontinued, and when stable, the monitors were discontinued. The recovery and extubation processes and the criteria that were checked for this RNR TBI model were similar to the ones described above for the CCI TBI model.

### 4.4. Data Analysis

The results of AA serum level measurements obtained after diffuse and focal TBI from the Development Dataset were combined to better mimic the heterogeneity of TBI in humans. The change in each AA concentration over time following TBI was evaluated for significant differences using the Mann–Whitney U test (SPSS v22, IBM, New York, NY, USA) because the data were not normally distributed for all the serum AAs. P-values <0.05 were considered statistically significant. As previously mentioned, the Development Dataset includes 25 pre-injury assessments, serving as healthy controls, and 25 post-TBI assessments (24 h post-TBI, *n* = 16 and 4-days post-TBI, *n* = 9). Correlation analyses between all AA concentrations in serum and tissue from the Development Dataset were performed, the corresponding 95% confidence ellipses for both sham and injured (1-day) groups were plotted, and the correlation coefficient (R^2^) and p-values were calculated.

A multivariate logistic regression analysis, coupled with the best-subset selection technique, was used to perform a thorough search of all possible subset sizes and combinations of AAs, evaluate the prediction ability of any AA subset to correctly distinguish between injured and uninjured serum data, and find a robust and optimal multi-AA TBI biomarker panel. A schematic summarizing the workflow of these steps is shown in Figure 3. The multivariate logistic regression (logit) model has the following expression:

(1) $$Logit\;\left(P\right)=\ln\left(\frac{P}{1-P}\right)=\beta_{0}+\beta_{1}X_{1}+\beta_{2}X_{2}+\ldots +\beta_{p}X_{p}$$

where P is the estimated probability of occurrence, in this study occurrence of TBI, $Logit\;\left(P\right)$ is the multivariate logistic regression model as a combination of p variables, $X_{1},\;X_{2},\ldots ,X_{p}$, which are AA levels in serum in this study, $\beta_{0}$ is a constant or intercept of the logit equation, and $\beta_{1},\;\beta_{2},\ldots ,\beta_{p}$ are the coefficients of the $X_{1},\;X_{2},\ldots ,X_{p}$ variables

The best-subset approach employs an exhaustive search of all possible subsets and then uses a criterion to choose the optimal subset. Different penalized-likelihood information criteria such as Akaike’s information criterion (AIC), Bayesian information criterion (BIC), and the Consistent AIC (CAIC) which consists of a goodness-of-fit term (l, maximum log-likelihood) plus a penalty term ($A_{n}p$), as expressed in the below formulation (Equation (2)) [29,38], can be coupled with logistic regression analysis to determine the best subset of variables in the regression model. These criteria provide a standardized way to balance sensitivity and specificity of the logit model and control overfitting.

(2) $$-2l+\;A_{n}p$$

where $A_{n}$ is a function of the sample size n (*n* = 50 in this study) and p is the size of subset of variables in the model (*p* = 1 to 17 in this study) and $A_{n}$ for AIC, BIC and CAIC criteria is n, ln(n), and ln(n)+1, respectively. When there are n variables (*n* = 50 in this study), the number of all possible subsets in the best subset selection approach is 2^17^ (131072 in this study) which includes C (p, pp)=pp!/(pp-p)!p! number of subsets with p variables (*p* = 1 to 17 and pp = 17 in this study) and pp is the total number of variables. For selection of the optimal subset of variables, AIC, BIC and CAIC criteria showed to have similar performance, but CAIC is preferred over AIC and BIC because it usually results in a more parsimonious regression model with the optimal and minimum number of variables, and less chance of overfitting [29]. Therefore, CAIC was used as the variable selection criterion in this study to determine the best subset of AAs and control overfitting. The regression model which resulted in the lowest CAIC represents the best fit model with optimal variables.

In addition to using the best subset selection technique, the multivariate logistic regression analysis was combined with repeated k-fold cross validation (CV) technique to reduce possible overfitting and dependency of the optimal fitted model to the Development Dataset and thus, possible bias in the selection of the AAs for the optimal multi-AA regression biomarker model [32]. Therefore, instead of using all 25 uninjured samples and 25 injured serum samples as the Development Dataset in a single analysis to find the optimal multi-AAs, the data was repeatedly split into k (k = 5) equal-sized sub samples, and the process of finding the best set of AAs was repeated for 25 iterations (5 repetitions of 5-fold CV). At each iteration of k-fold CV, k-1 subsamples were combined and used as a training dataset to fit the models, using all 2^17^ possible subsets which include subsets with 1 to 17 AAs, and CAIC criterion for all models were calculated. The best 1-to-17-AA models which resulted in smallest CAIC values were identified. The outputs of each iteration were: a) 17 values of CAIC, and b) the variables included in the best 1-to-17-AA models based on the CAIC criterion. At each iteration of k-fold CV, the remaining sub-sample from the initial k subsamples was left out to be used as testing dataset later for prediction capability evaluation. For every k-fold CV, this procedure was repeated k times until all k sub-samples were used in the testing dataset once. The entire process of 5-fold CV was repeated 5 times resulting in 25 iterations. The averages and standard deviations of the CAIC variable selection criterion over the 25 iterations for 1- to 17-AA subsets were then calculated and plotted to determine the optimal number (m) of AA variables which resulted in the best regression model with lowest average CAIC criteria value for the entire 5 repetition 5-fold CV process. The next step was to determine the variables included in the optimal m-AA model. This combination of m variables included the ones that consistently or repeatedly were selected as the best m-AA subset over the 25 iterations of the 5 repetition 5-fold CV process. The outputs of this step were the optimal number and combination of the AAs in the final multi-AA biomarker model.

The TBI diagnostic/prediction capability of the optimal subset of AAs, identified using the rigorous subset selection procedures, was evaluated in four ways. First, the optimal subset of AAs was used to develop a multivariate logit model using the entire Development Dataset (25 pre-injury samples and 25 post-TBI samples) and the optimal ROC threshold value (optimizing specificity and sensitivity) of the generated regression model was determined. Then the determined optimal ROC threshold value and the measures of predictive capability, sensitivity (S), specificity (SP), and overall prediction accuracy rate (PAR) on the same dataset using the developed logit model were calculated as follows [32]:

(3) $$S=Sensitivity=\frac{TPositive}{Positive}$$

(4) $$SP=Specificity=\frac{TNegative}{Negative}$$

(5) $$PAR=Overall\;Prediction\;Accuracy\;Rate=\frac{TNegative+TPositive}{Negative+Positive}$$

where Positive is the number of TBI subjects, Negative is the number of non-injured/healthy control cases (samples from pre-TBI in this study), and TPositive and TNegative are the number of cases correctly identified as TBI and non-injured, respectively. For more rigorous and realistic evaluation of prediction performance, at each iteration of the 5 repetition 5-fold process previously performed, the logit m-AA model was developed on the training dataset, the optimal ROC threshold value was determined, and S, SP, and PAR values were calculated for the testing dataset. The average and standard deviation of S, SP, PAR and the threshold value were calculated and reported. Then the m-AA logit model developed in the first approach, in which the entire Development Dataset (n=50) was used, was applied to the Validation Dataset (4 uninjured and 5 RNR 24hrs-post TBI) and S, SP, and PAR were calculated to determine the prediction/diagnostic capability of the optimal m-AA model in an entirely separate and unseen dataset. Finally, this optimal m-AA biomarker model was applied to the Exploration Dataset (5 CCI and 5 RNR 8 days post TBI) and S value was calculated to evaluate of the prediction capability of the developed optimal multivariate biomarker model to diagnose TBI over extended time following injury (8 days). All data analyses were performed in MATLAB (V. R2015 MathWorks, Columbia, MD, USA).

## Figures and Tables

**Table 1:** Details of the samples from the three datasets used in this study.

Development Dataset	Validation Dataset	Exploration Dataset
25 piglets, 50 serum samples including: 25 pre-TBIs (controls) 12 focal post-TBIs (*n* = 8 at 24-h post-TBI, *n* = 4 at 4-days post-TBI) and 13 diffuse post-TBIs (*n* = 8 at 24-h post-TBI, *n* = 5 at 4-days post-TBI)	9 piglets, 9 serum and right cortex brain tissue samples including: 4 shams and 5 diffuse post-TBIs (at 24-h post-TBI)	10 piglets, 10 serum samples including: 5 diffuse post-TBIs (at 8-days post-TBI) and 5 diffuse post-TBIs (at 8-days post-TBI)

**Table 2:** The optimal multivariate regression model using serum concentration of glycine, taurine, and ornithine.

Coefficients	Estimate	Standard Error	Z-Value	P-value
(Intercept)	7.778	2.342	3.321	0.001
GLY	−0.013	0.004	−3.307	0.001
TAU	−0.064	0.030	−2.147	0.032
ORN	0.065	0.024	2.683	0.007

**Table 3:** The results of the ROC curve analysis using the optimal 3-AA regression model and each of the univariate AA biomarker.

Biomarker	AUC	ROC Optimal Threshold
GLY-TAU-ORN regression model	0.92	0.439 (logit score-Table 2)
ALA	0.58	295 nm/mL
ARG	0.53	145 nm/mL
ASN	0.58	30 nm/mL
CIT	0.55	56 nm/mL
GLN	0.57	286 nm/mL
GLU	0.53	168 nm/mL
GLY	0.79	729 nm/mL
IL	0.64	66 nm/mL
LEU	0.56	81 nm/mL
LYS	0.56	76 nm/mL
ORN	0.52	58 nm/mL
PHE	0.54	48 nm/mL
SER	0.68	85 nm/mL
TAU	0.65	48 nm/mL
THR	0.59	130 nm/mL
TYR	0.61	34 nm/mL
VAL	0.60	141 nm/mL

**Table 4:** Prediction capability of the 3-AA logistic regression biomarker model.

	Sensitivity	Specificity	Overall Accuracy Rate
Development set	80%	92%	86%
Validation set	100%	50%	78%
Exploration set	80%		
Five repeated five-fold CV (Avg ± SD)	82% ± 18%	74% ± 20%	79% ± 10%

**Table 5:** The correlation coefficients (R^2^) between serum and brain tissue concentrations of seventeen amino acids for both healthy/sham and TBI group (24-h post-TBI).

AA	Correlation Analysis	
	Sham	Injured-1Day
	R^2^	R^2^
ALA	−0.92	0.94
ARG	0.90	0.92
ASN	−0.04	0.23
CIT	0.92	0.32
GLN	0.76	−0.78
GLU	−0.01	0.18
GLY	0.57	0.46
IL	0.90	0.63
LEU	0.78	0.65
LYS	0.94	0.64
ORN	0.99	0.88
PHE	0.64	−0.06
SER	0.44	0.37
TAU	0.68	−0.27
THR	0.95	0.24
TYR	0.55	0.86
VAL	0.74	0.80

## Abbreviations

TBI	Traumatic brain injury
Logit	Logistic regression analysis
S	Sensitivity
SP	Specificity
PAR	Prediction accuracy rate
ROC	Receiver operating characteristic
CAIC	Consistent Akaike’s information criterion
AIC	Akaike’s information criterion
BIC	Bayesian information criterion
CV	Cross validation
RNR	Rapid non-impact rotational
CCI	Controlled cortical impact
AA	Amino acid
ALA	Alanine
ARG	Arginine
ASN	Asparagine
CIT	Citrulline
GLN	Glutamine
GLU	Glutamate
GLY	Glycine
IL	Isoleucine
LEU	Leucine
LYS	Lysine
ORN	Ornithine
PHE	Phenylalanine
SER	Serine
TAU	Taurine
THR	Threonine
TYR	Tyrosine
VAL	Valine
KNN	k-nearest-neighbors
LDA	Linear discriminant analysis
